# Nonsyndromic Gingival Fibromatosis: A Rare Case Report

**DOI:** 10.5005/jp-journals-10005-1521

**Published:** 2018-06-01

**Authors:** Mahima Gandhi, Sandeep Tandon, Meenakshi Sharma, Akshat Vijay

**Affiliations:** 1 Resident (Final Year), Department of Pediatric and Preventive Dentistry, Government Dental College, Jaipur, Rajasthan, India; 2 Senior Professor and Head, Department of Pediatric and Preventive Dentistry, Government Dental College, Jaipur, Rajasthan, India; 3 Assistant Professor, Department of Pediatric and Preventive Dentistry, Government Dental College, Jaipur, Rajasthan, India; 4 Resident (Final Year), Department of Orthopedics, Jawaharlal Nehru Medical College Ajmer, Rajasthan, India

**Keywords:** Attached gingiva, Gingivectomy, Hereditary gingival fibromatosis, Mixed dentition.

## Abstract

Hereditary gingival fibromatosis (HGF) is an uncommon gingival disease of attached gingiva, which is manifested as localized or generalized form. The HGF inheritance is transmitted through both autosomal dominant and recessive modes. Here, we are discussing a rare case report of an 8-year-old child with gingival fibromatosis in mixed dentition, which caused damage to his speech, mastication, and esthetics and led to significant change in his facial profile.

The patient noticed that the gingival enlargement was simultaneous with deciduous dentition eruption and gradually covered entire dentition. Gingival enlargement covered all teeth anteriorly and posteriorly and only occlusal surfaces were visible. The enlarged tissue was resected by the external bevel gingivectomy under general anesthesia arch wise.

The postoperative healing was satisfactory, uneventful, and there was significant change in patient’s esthetics. Patient has been kept on regular recall visits.

**How to cite this article:** Gandhi M, Tandon S, Sharma M, Vijay A. Nonsyndromic Gingival Fibromatosis: A Rare Case Report. Int J Clin Pediatr Dent 2018;11(3):250-253.

## CASE REPORT

An 8-year-old male child reported to the Department of Pediatric and Preventive Dentistry, Government Dental College and Hospital, Jaipur, Rajasthan, India, with chief complaint of gingival swelling and absence of teeth, which caused damage to his speech, mastication, and esthetics and led to a significant change in his facial profile due to severe gingival enlargement.

The patient noticed that the gingival enlargement was simultaneous with deciduous dentition eruption and gradually covered entire dentition. Gingival enlargement covered all teeth anteriorly and posteriorly, and only occlusal surfaces were visible.

**Figs 1A and B:**
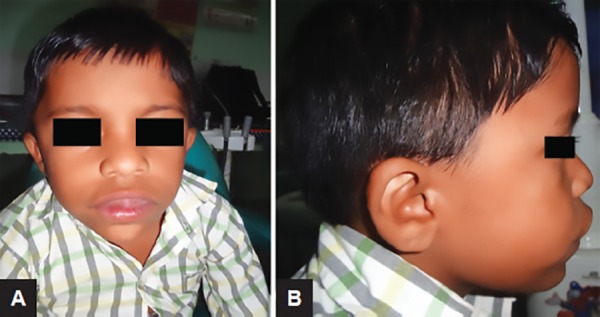
Extraoral profile view

Extraoral examination of patient was done with convex profile, lips incompetency, and enlarged tissue was protruding out of the mouth (Fig. 1).

**Fig. 2:**
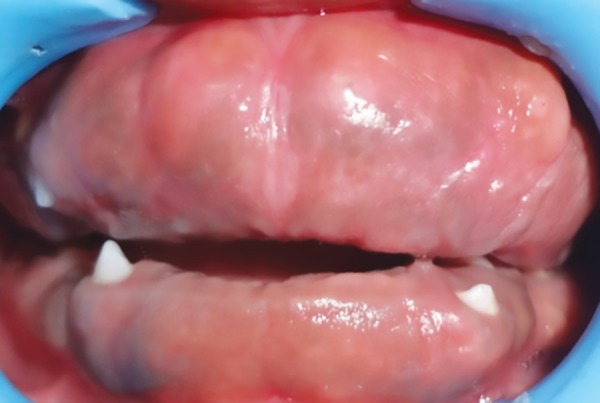
Intraoral view

**Figs 3A and B:**
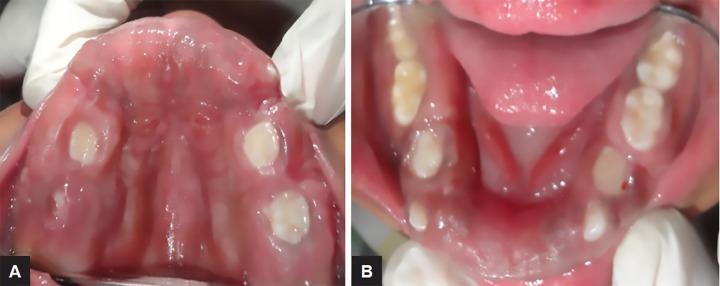
Intraoral view of maxillary and mandibular arch

Intraoral examination of generalized gingival enlargement including both mandibular and maxillary arches and vestibule was recorded (Figs 2 and 3). The tissue was pink, leathery in consistency, and firm on palpation and covered entire dentition. There were no signs of ulceration on enlarged tissue due to mastication forces.

**Fig. 4:**
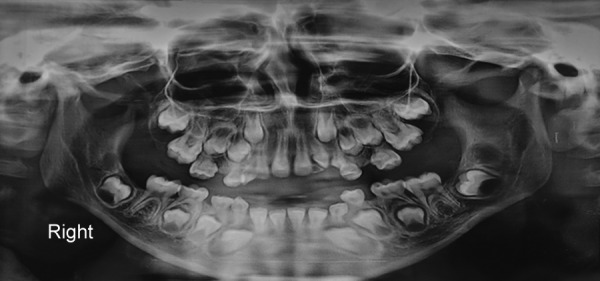
Orthopantomogram

A scanty amount of food debris and plaque was present on the posterior teeth, but there were no signs of inflammation present. Due to excessive gingival growth, positions of teeth were difficult to determine. However, the panoramic radiograph showed normal bone height and tooth positioning (Fig. 4). Routine blood investigations were done and values were found within normal limit.

**Fig. 5:**
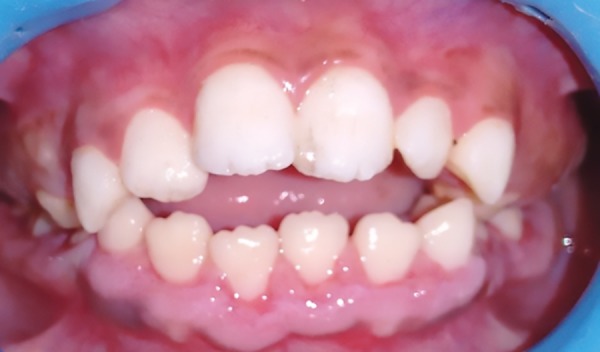
Postoperative frontal view

Based on provisional diagnosis, an incisional biopsy was done and section was evaluated. The patient’s medical history was nonsignificant for any hormonal changes or drug-induced gingival enlargement and exhibited no signs of mental retardation or hypertricho-sis. His parents did not reveal any evidence of gingival overgrowth.

The enlarged tissue was excised by the external bevel gingivectomy under general anesthesia arch wise. Periodontal dressing was given and antibiotics were prescribed. Postoperatively, periodontal dressing was placed on the surgical site and was covered with custom-made acrylic stent. Oral hygiene instructions were given. The histological evaluation revealed moderately dense to highly dense collagenous connective tissue with collagen bundles arranged in an irregular manner.

The connective tissue was relatively avascular with scanty inflammatory cell infiltrate. The overlying epithelium was hyperplastic with enlarged rete ridges. This was suggestive of gingival fibromatosis (Fig. 5). Postsurgical healing was satisfactory. Patient was recalled after 1 week for periodontal dressing removal and was on follow-up after 2 weeks, and at 1- and 3-month intervals for evaluation.

**Figs 6A and B:**
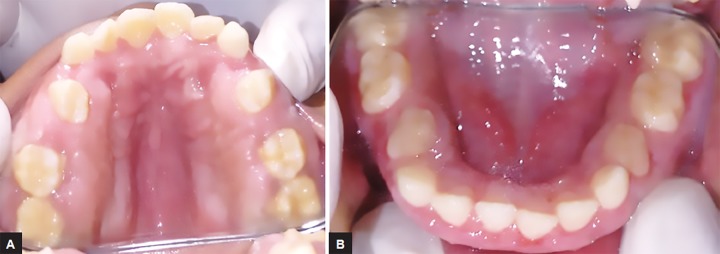
Postoperative intraoral view

**Figs 7A and B:**
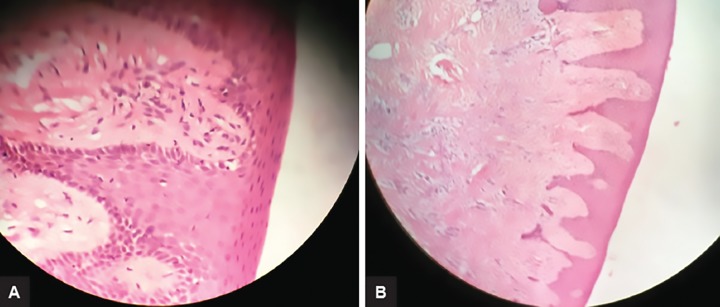
Histological picture under 40* and 10* magnification

Postsurgically, his facial profile improved drastically and the parents were satisfied with the results (Figs 6 and 7).

## DISCUSSION

Hereditary gingival fibromatosis, also quoted as idiopathic gingival hyperplasia, is a type of gingival enlargement. It is an unusual disease of the gingiva characterized by firm, enlarged gingival tissue that covers almost anatomic tooth crowns.

The onset of the disease is frequent during the eruption of the permanent incisors,^1^ but many reports also specified involvement of the deciduous dentition.^2^ It is classified as a nonplaque-induced gingival lesion caused by numerous genetic disorders.^3^ The HGF may occur as an isolated disorder, but it also has been reported to be linked with many syndromes and other abnormalities.^4^

Case reports have linked HGF with Murray-Puretic-Drescher syndrome (juvenile hyaline fibromatosis), Zimmerman-Laband syndrome (ear, nose, bone, and nail defects), Cowden syndrome (multiple hamartoma, hypopigmentation), Prune belly syndrome (absence of one or more layers of abdominal muscles).^5^ The HGF has both autosomal dominant and recessive modes of inheritance.

Gingival enlargement, defined as an increase or overgrowth in the size of the gingiva, is a common feature in gingival diseases and may be caused by array of factors.^6^ Etiologic features include inflammation, systemic diseases, medications, and neoplastic tissue changes. Based on the American Academy of Periodontology 1999 Classification of Periodontal Diseases and Conditions, the tissue enlargement involved both the attached and marginal gingiva^7^; it can be localized or generalized. The degree of enlargement can be classified as follows^8^:

- Grade 0: No gingival enlargement.
- Grade I: Enlargement confined to interdental papilla.
- Grade II: Enlargement involving papilla and marginal gingivae.
- Grade III: Enlargement covering three quarters or more of the crown.

The HGF is classified into two types, symmetric and nodular. The symmetric type is featured by uniform enlargement of gingiva.

The symmetric type is the most frequent type, while the nodular type is characterized by the presence of multiple tumors in dental papillae; however, combination of either types does occur. The localized form usually affects the maxillary molar and tuberosity area, particularly on the palatal surface. The symmetric type is mostly unilateral and has generalized or localized form. In severe involvement, teeth are almost completely covered.^9^

The onset of gingival overgrowth mostly coincides with the eruption of the permanent dentition or with the eruption of the primary dentition. Rarely, it can also be present at birth.^10^ The HGF has not been reported in edentulous patients. Hence, the presence of dentition is necessary for HGF to occur. The indicated treatments differ according to the degree of severity.

In minimal enlargement cases, for good oral health maintenance, oral prophylaxis and home care may be sufficient. As the tissue excess increases, appearance and functional impairment mandate the need for surgical intervention.^11^ Because of the severity of the involvement in this case, favored treatment was gingivectomy under general anesthesia.

The various treatment options for HGF include surgery, electrocautery, and carbon dioxide laser. Nowadays, carbon dioxide laser is the treatment of choice.^12^ If carbon dioxide laser is not available, the conventional external bevel gingivectomy is effective for removing large quantities of gingival tissue, especially in the absence of attachment loss and false pocketing.^11^ Emerson^13^ recommended the best times to be at the ages of 3, 6, and 12 years.

Prognosis of HGF depends on oral prophylaxis and home care.

## CONCLUSION

Hereditary gingival fibromatosis is an uncommon disease of attached gingiva characterized by different degrees of overgrowth. Surgical intervention is necessary for esthetic and functional demand, although recurrence cannot be determined.

Patient was kept under review and there was no occurrence of gingival overgrowth on a 6-month follow-up.

## CLINICAL SIGNIFICANCE

Timely removal of fibrous tissue in child promotes good nutrition through proper chewing, assists in speech development, builds patient confidence by enhancing appearance and provides way for permanent teeth eruption.
